# Correction: Hepatitis C Virus Core Protein Down-Regulates p21^Waf1/Cip1^ and Inhibits Curcumin-Induced Apoptosis through MicroRNA-345 Targeting in Human Hepatoma Cells

**DOI:** 10.1371/journal.pone.0181299

**Published:** 2017-07-07

**Authors:** Tzu-Yue Shiu, Shih-Ming Huang, Yu-Lueng Shih, Heng-Cheng Chu, Wei-Kuo Chang, Tsai-Yuan Hsieh

In Fig 4B, the 5^th^ and 7^th^ fluorescent microscopy images for HA-Core191 + miR-345 inhibitor (10nM) and HA-Core173 + miR-345 inhibitor (10nM) are duplicates of each other. The authors have provided a new set of cell images form the same experiment done at a different time for Fig 4B. Please see the correct Fig 4 and its caption below.

**Fig 4:**
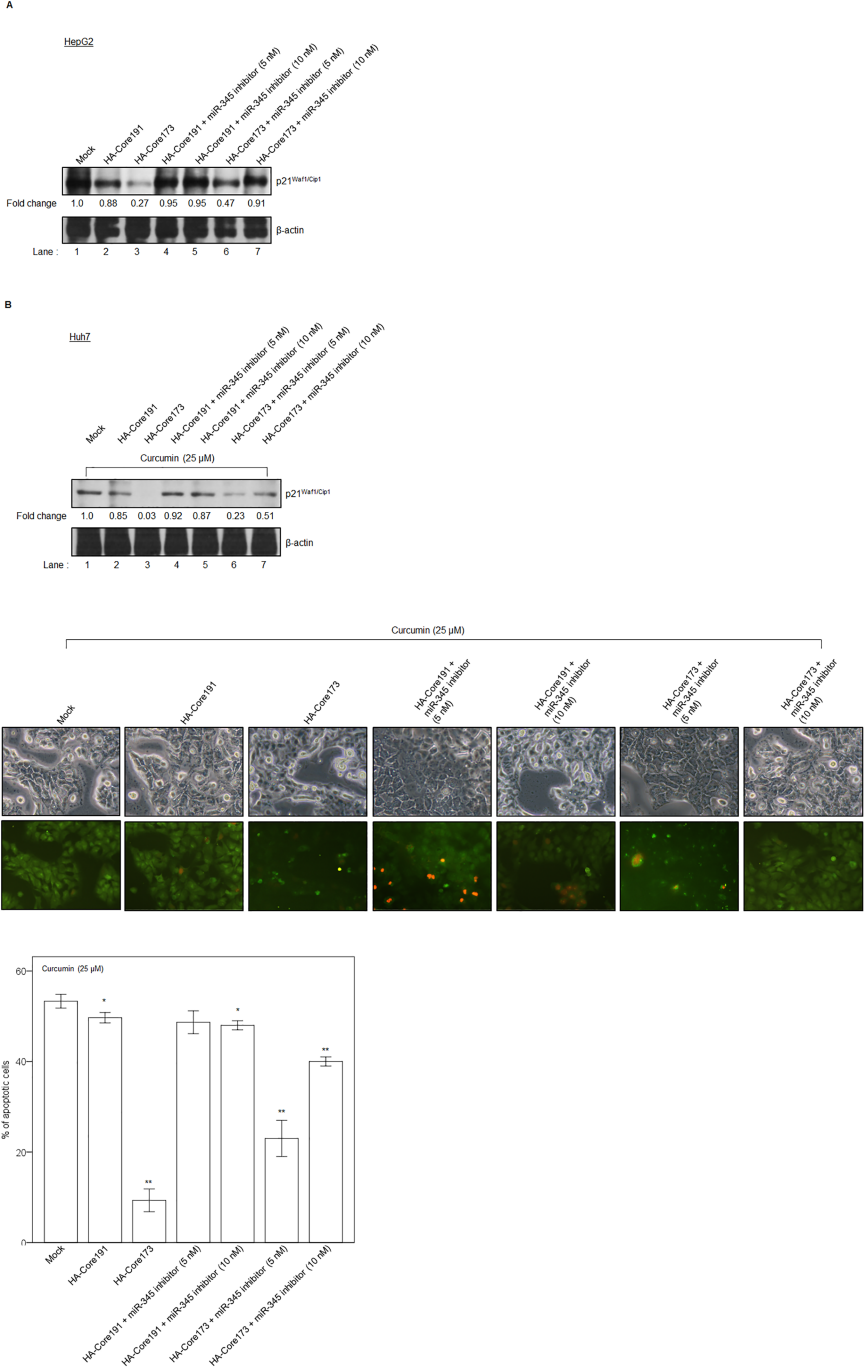
HCV core-induced microRNA-345 inhibits *p21*^*Waf1/Cip1*^ gene expression in HepG2 cells and curcumin-stimulated Huh7 cells. (**A**) HCV core (amino acid 1–191 or 1–173)-expressing HepG2 cells were transfected with miR-345 inhibitor (5 nM or 10 nM). At 24 hours after transfection, *p21*^*Waf1/Cip1*^ gene expression at protein level was analyzed by Western blotting. β-actin served as an internal control. (**B**) HCV core (amino acid 1–191 or 1–173)-expressing Huh7 cells in response to curcumin stimulation were transfected with miR-345 inhibitor (5 nM or 10 nM). At 24 hours after transfection, *p21*^*Waf1/Cip1*^ gene expression at protein level was analyzed by Western blotting. β-actin served as an internal control (upper panel). Apoptosis was analyzed by fluorescence microscopy (middle panel) and FACS Calibur (lower panel) using Annexin V-FITC apoptosis assay. Original magnifications ×200. Cells from early apoptotic stage were stained with annexin V-FITC, and appeared green. Cells from late apoptotic stage were stained with both annexin V-FITC and PI, and merged to be yellow. Data was shown as the means ± S.D. from triplicate experiments. *P<0.05, **P<0.001.

The authors would like to clarify that for Fig 3C, although the last two FACS Calibur images appear to have identical sections, these images are acquired directly from the computer machine in their original form. They have provided the images in S1 Dataset.

## Supporting information

S1 Dataset Raw FACS Calibur images for Fig 3C. (ZIP)
