# Supplementary figures and images for: Correction: Hepatitis C Virus Core Protein Down-Regulates p21Waf1/Cip1 and Inhibits Curcumin-Induced Apoptosis through MicroRNA-345 Targeting in Human Hepatoma Cells
Source: PLoS One. 2017 Jul 7;12(7):e0181299. doi: 10.1371/journal.pone.0181299 (PMC5501671; doi:10.1371/journal.pone.0181299)

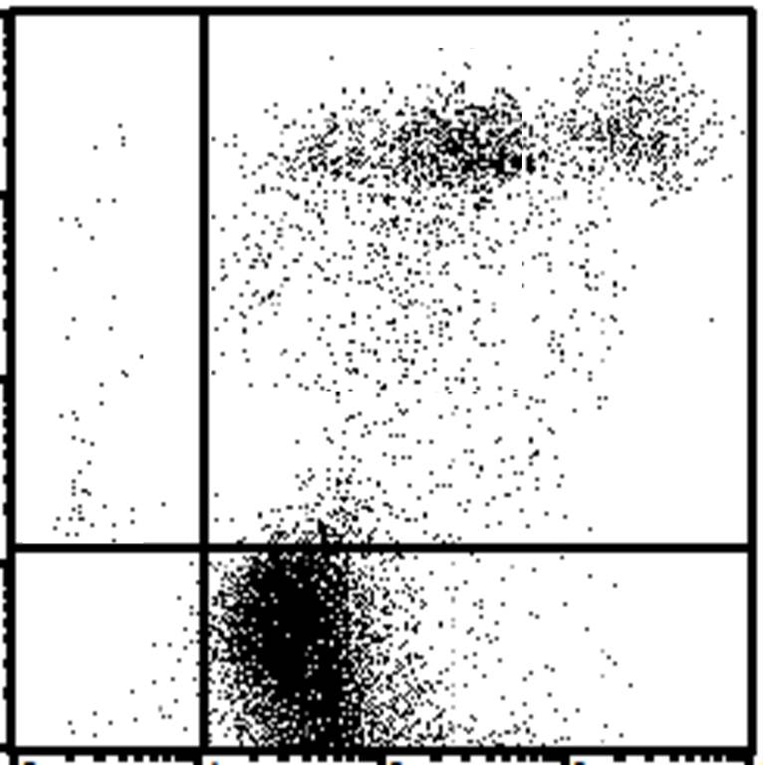

Supplement: S1 Dataset — (ZIP) [file pone.0181299.s001.zip › 3C1 Curcumin+mimic (0nM).tif]

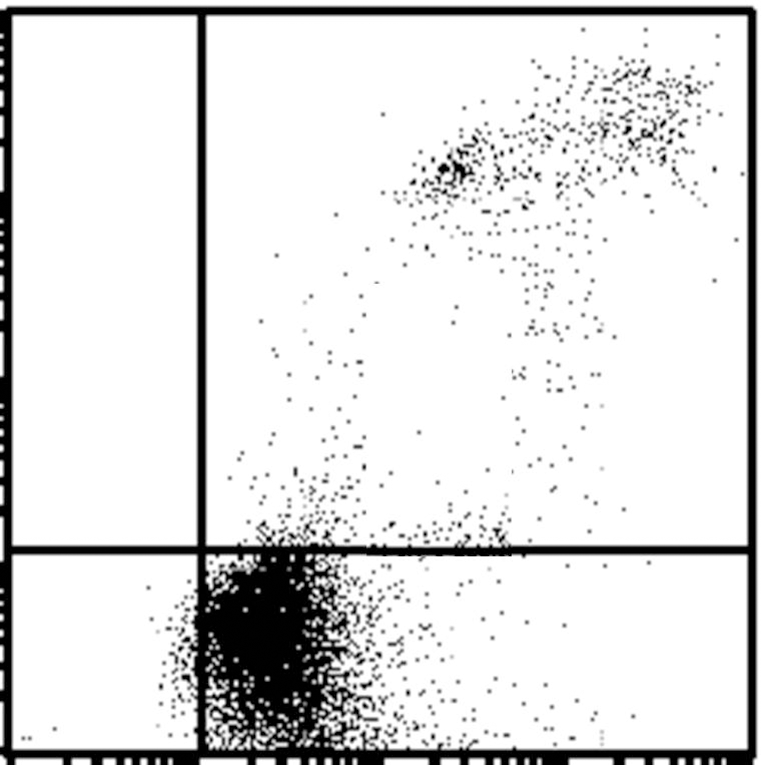

Supplement: S1 Dataset — (ZIP) [file pone.0181299.s001.zip › 3C2 Curcumin+mimic (5nM).tif]

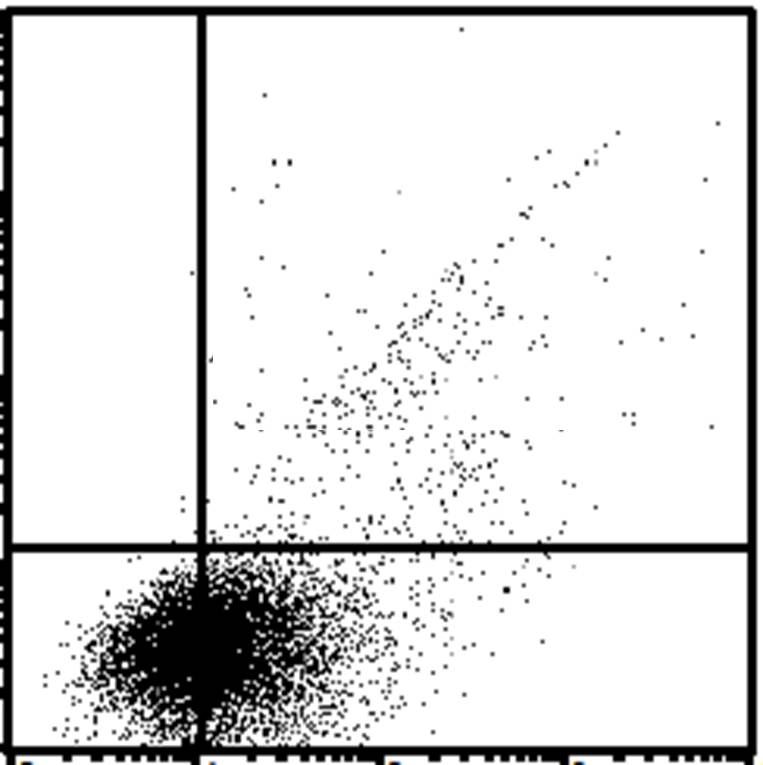

Supplement: S1 Dataset — (ZIP) [file pone.0181299.s001.zip › 3C3 Curcumin+mimic (10nM).tif]

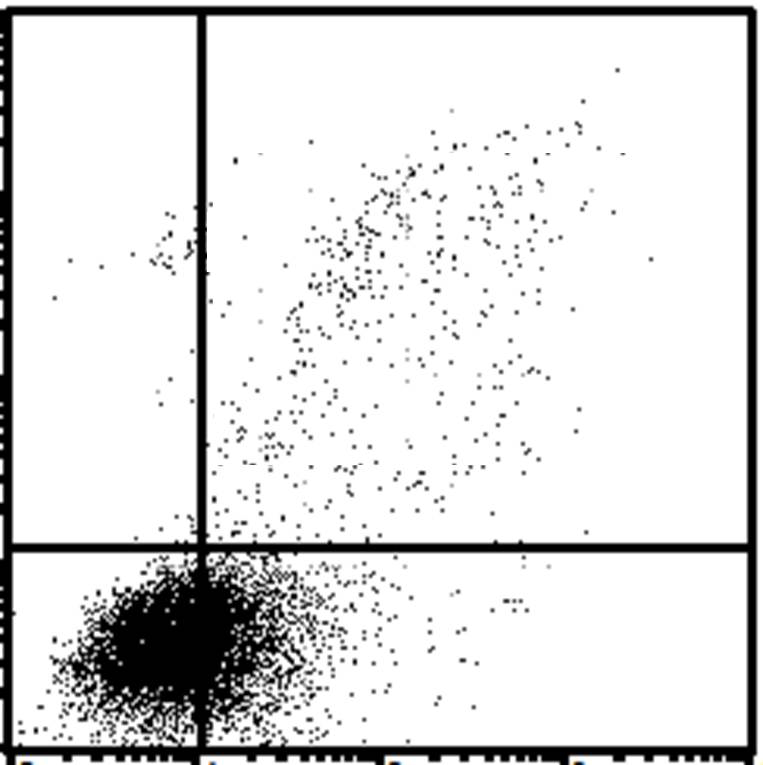

Supplement: S1 Dataset — (ZIP) [file pone.0181299.s001.zip › 3C4 Curcumin+mimic (25nM).tif]

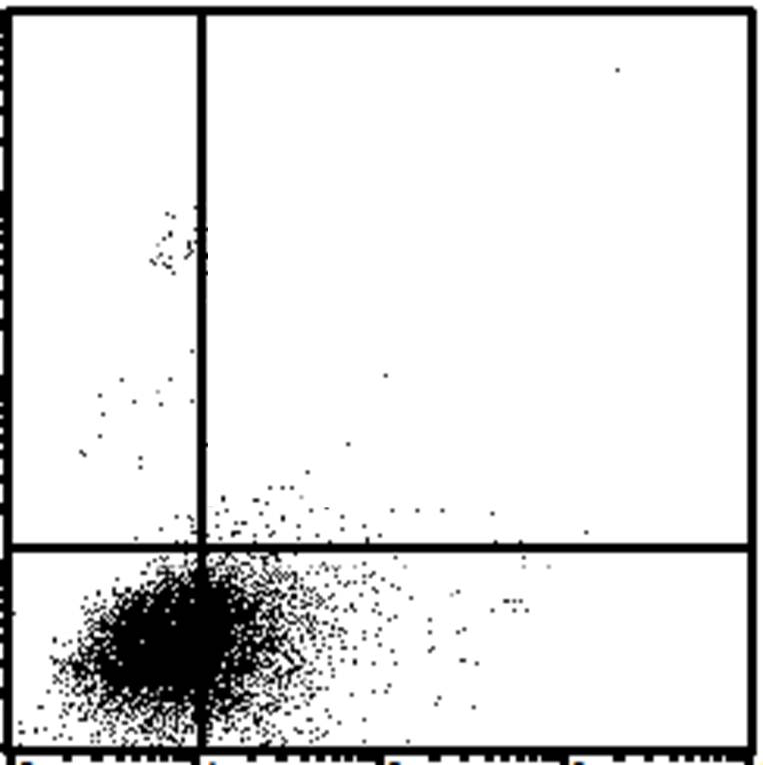

Supplement: S1 Dataset — (ZIP) [file pone.0181299.s001.zip › 3C5 Curcumin+mimic (50nM).tif]
